# 5-Amino-1*H*-benzimidazole-2(3*H*)-thione: mol­ecular, crystal structure and Hirshfeld surface analysis

**DOI:** 10.1107/S2056989022000792

**Published:** 2022-01-28

**Authors:** Dilnoza Rakhmonova, Lobar Gapurova, Surayyo Razzoqova, Shakhnoza Kadirova, Batirbay Torambetov, Zukhra Kadirova, Svitlana Shishkina

**Affiliations:** a National University of Uzbekistan named after Mirzo Ulugbek, 4 University St, Tashkent, 100174, Uzbekistan; b Uzbekistan–Japan Innovation Center of Youth, University Street 2B, 100095, Tashkent, Uzbekistan; c State Scientific Institution "Institute for Single Crystals" of National Academy of Sciences of Ukraine, 60 Nauky ave., 61001 Kharkiv, Ukraine

**Keywords:** mol­ecular structure, crystal structure, 5-amino-1*H*-benzimidazole-2(3*H*)-thione, hydrogen bond, Hirshfeld analysis, periodic calculations

## Abstract

The title compound, C_7_H_7_N_3_S, which has potential biological activity, can be used as a ligand in metal complexation. This compound exists as the thione tautomer in the crystal phase, which is confirmed by the study of its mol­ecular structure. The amino group has pyramidal configuration. In the crystal phase, the two independent mol­ecules in the asymmetric unit form tetra­mers as a result of N—H⋯S hydrogen bonds. These tetra­mers are linked by N—H⋯N hydrogen bonds, forming chains/tubes in the [010] direction. The Hirshfeld surface analysis showed that the highest contribution to the total surface is provided by H⋯H inter­actions as well as S⋯H/H⋯S and C⋯H/H⋯C contacts associated with *X*—H⋯S hydrogen bonds and *X*—H⋯C(π) inter­actions.

## Abstract 2

The mol­ecular and crystal structures of the anhydrous form of 5-amino-1*H*-benzimidazole-2(3*H*)-thione were determined. Hirshfeld surfaces and fingerprint plots were studied.

## Chemical context

Benzimidazoles belong to an important class of heterocyclic compounds because of their wide spectra of biological activity. In particular, benzimidazole derivatives are known to possess anti­bacterial (Chkirate *et al.*, 2020 ▸), anti­microbial (Alam *et al.*, 2014 ▸), anti­tumor (Kharitonova *et al.*, 2018 ▸; Galal *et al.*, 2010 ▸), anti-inflammatory (Rathore *et al.*, 2017 ▸), anti­oxidant (Anastassova *et al.*, 2017 ▸), anthelmintic (Kenchappa *et al.*, 2017 ▸), anti­fungal and cytotoxic (Leila *et al.*, 2019 ▸) activity. They are also important as starting materials for terminal alkyne cyclo­trimerization reactions (Xi *et al.*, 2013 ▸) and are used as highly active catalysts for ethyl­ene oligomerization (Haghverdi *et al.*, 2018 ▸). The synthesis of 2-amino-1,3-benzimidazole-2-thione has been reported, prepared by first treating *o*-phenyl­enedi­amine CS_2_ in the presence of KOH under microwave irradiation to give the inter­mediate 1,3-benzimidazole-2-thione. Nitration of the inter­mediate followed by reduction of the nitro group with iron powder and concentrated hydro­chloric acid gave 2-amino-1,3-benzimidazole-2-thione in a moderately good yield (Samanta *et al.*, 2013 ▸; Ahamed *et al.*, 2013 ▸). Taking into account the possible biological activity of the obtained compound, it is important to study its mol­ecular and crystal structures.

## Structural commentary

**Figure 1:**
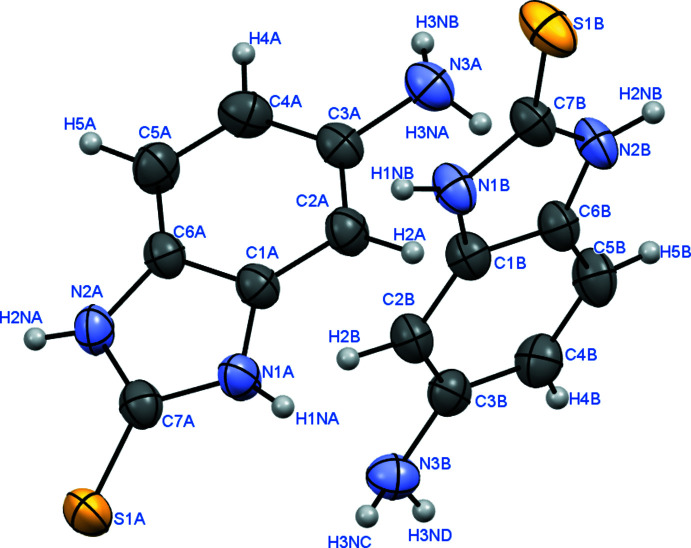
Mol­ecular structures of mol­ecules *A* and *B* showing the atom-labelling scheme. Displacement ellipsoids are drawn at the 50% probability level.

Two independent mol­ecules (*A* and *B*) comprise the asymmetric unit of the title compound (Fig. 1 ▸). The mol­ecules slightly differ from each other in their degree of planarity: all non-hydrogen atoms lie in the same plane with an accuracy of 0.05 Å in mol­ecule *A* and with an accuracy of 0.02 Å in mol­ecule *B*. Analysis of the mol­ecular structure revealed that the C=S tautomer is found in the crystal, as confirmed by the length of the C7—S1 bond [1.68*7* (3) Å in mol­ecule *A* and 1.684 (3) Å in mol­ecule *B*], the equal lengths of the C7—N1 and C7—N2 bonds [1.34*5* (3) and 1.347 (3) Å in mol­ecule *A* and 1.351 (3) and 1.349 (3) Å in mol­ecule *B*] and the localization of hydrogen atoms at all the nitro­gen atoms from the electron-density difference maps. The amino groups in both mol­ecules are pyramidal, the sum of the bond angles centered at the nitro­gen atom is 331.5° in mol­ecule *A* and 340.9° in mol­ecule *B*. 
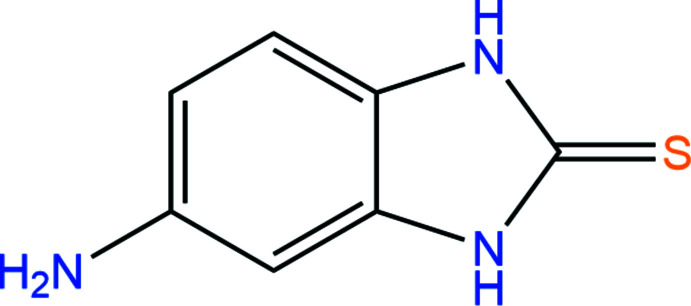


## Supra­molecular features

**Figure 2:**
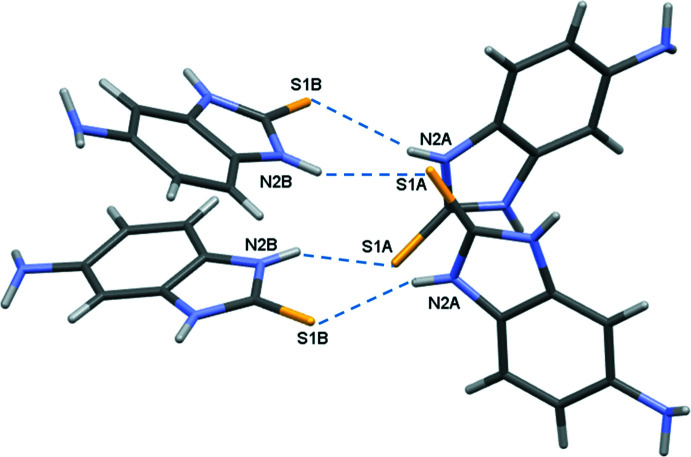
Tetra­mer of mol­ecules *A* and *B* formed by N2*A*—H2*NA*⋯S1*B* and N2*B*—H2*NB*⋯S1*A* hydrogen bonds.

**Figure 3:**
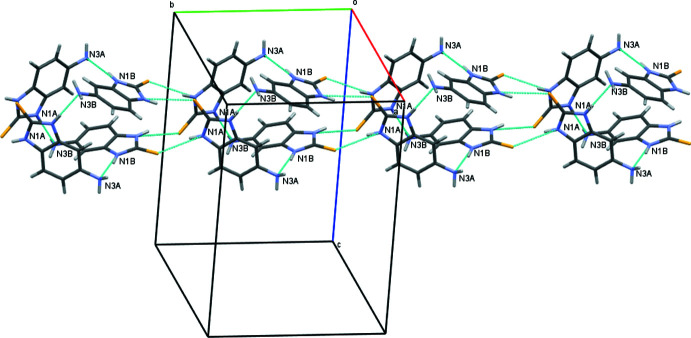
Chain/tube of tetra­mers linked by N1*A*⋯H1*NA*—N3*B* and N1*B—*H1*NB*⋯N3*A* hydrogen bonds.

In the crystal, the mol­ecules form tetra­mers as a result of the N2*A*—H2*NA*⋯S1*B* and N2*B*—H2*NB*⋯S1*A* hydrogen bonds (Fig. 2 ▸, Table 1 ▸). The tetra­mers are linked by N1*A*⋯H1*NA*—N3*B* and N1*B—*H1*NB*⋯N3*A* hydrogen bonds, forming a tube in the [010] direction (Figs. 3 ▸ and 4 ▸). Adjacent tubes are connected by weaker N—H⋯C(π), C—H⋯S, N—H⋯S and C—H⋯C(π) inter­actions (Table 1 ▸).

## Hirshfeld surface analysis

**Figure 5:**
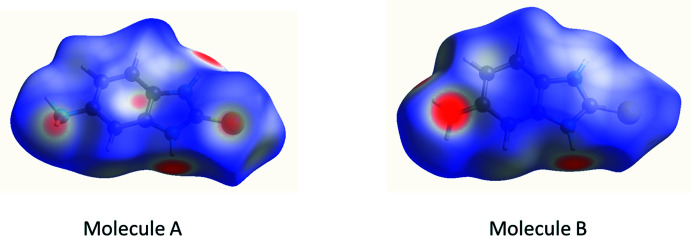
Hirshfeld surfaces mapped over *d*_norm_ calculated for mol­ecules *A* and *B*.

One of the modern methods for analysing inter­molecular inter­actions is Hirshfeld surface analysis (Spackman & Jayatilaka, 2009 ▸; Turner *et al.*, 2017 ▸), which allows analysis of the inter­actions between mol­ecules in a qu­anti­tative manner. The Hirshfeld surfaces of mol­ecules *A* and *B* mapped over *d*_norm_ proved to be very similar (Fig. 5 ▸). The red spots indicating strong inter­actions are found at both hydrogen atoms of the NH fragments as well as in the area of the nitro­gen lone pair of the amino group. In addition, red spots are seen at the sulfur atom.

**Figure 6:**
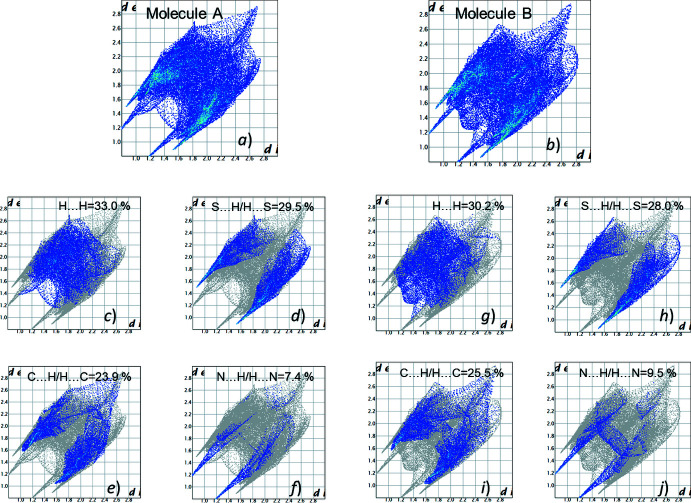
Two-dimensional Hirshfeld fingerprint plot of all contacts for mol­ecules *A* (*a*) and *B* (*b*) and those delineated into H⋯H (*c*, *g*), S⋯H/H⋯S (*d*, *h*), C⋯H/H⋯C (*e*, *i*) and N⋯·H/H⋯N (*f*, *j*) contacts.

Analysis of the fingerprint plots showed the presence of strong inter­molecular inter­actions indicated as sharp spikes (Fig. 6 ▸ *a*, 6*b*). The most significant contribution to the total Hirshfeld surface is provided by H⋯H inter­actions in both mol­ecules (Fig. 6 ▸ *c*, 6*g*). The contributions of S⋯H/H⋯S and C⋯H/H⋯C inter­actions associated with *X*—H⋯S and *X*—H⋯C (π) hydrogen bonds are similar (Fig. 6 ▸ *d*–*i*). Surprisingly, the contribution of N⋯H/H⋯N inter­actions proved to be the lowest (Fig. 6 ▸ *f*, 6*j*). It may be explained by the participation of the nitro­gen lone pair in hydrogen bonding as a proton acceptor.

## Database survey

**Figure 7:**
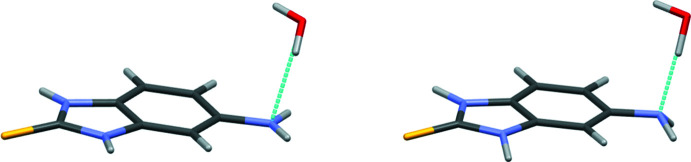
Configuration of the amino group in the structure of ODAXID calculated from the experimental data (Hadjikakou & Light, 2016 ▸) and obtained after optimization with a periodic boundary.

A search of the Cambridge Structural Database (Version 5.42, update of November 2020; Groom *et al.*, 2016 ▸) revealed the structure of the monohydrate of the title compound (ODAXID; Hadjikakou & Light, 2016 ▸). It should be noted that the amino group was refined as planar in this structure. However, analysis of the inter­molecular inter­actions showed that this amino group participates in a hydrogen bond with the hydrate water mol­ecule as a proton acceptor. Such a hydrogen bonding has to result in pyramidalization of the amino group. To check this presumption, we have optimized the ODAXID structure with a periodic boundary using the PBE functional (Adamo & Barone, 1999 ▸) within *Quantum Espresso* (Giannozzi *et al.*, 2009 ▸, 2017 ▸). The unit-cell parameters were fixed while the mol­ecular structures of both mol­ecules found in the asymmetric unit were optimized. The result of this optimization shows that the amino group has to be pyramidal (Fig. 7 ▸).

## Crystallization

5-Amino-1*H*-benzimidazole-2(3*H*)-thione was purchased from Sigma-Aldrich for use as a ligand in complexation with metals. The reaction of the title compound with nickel acetate in an aqueous alcoholic medium did not result in complex formation. The formed colourless needle-like crystals proved to be anhydrous form of the ligand with *T*_melt._ = 513–517 K.

## Refinement

Crystal data, data collection and structure refinement details are summarized in Table 2 ▸. All the hydrogen atoms were located in difference-Fourier maps and refined using an isotropic approximation.

## Supplementary Material

Crystal structure: contains datablock(s) I. DOI: 10.1107/S2056989022000792/ex2051sup1.cif

Structure factors: contains datablock(s) I. DOI: 10.1107/S2056989022000792/ex2051Isup2.hkl

Supporting information file. DOI: 10.1107/S2056989022000792/ex2051Isup3.cml

CCDC reference: 2143895

Additional supporting information:  crystallographic information; 3D view; checkCIF report

## Figures and Tables

**Figure 4:**
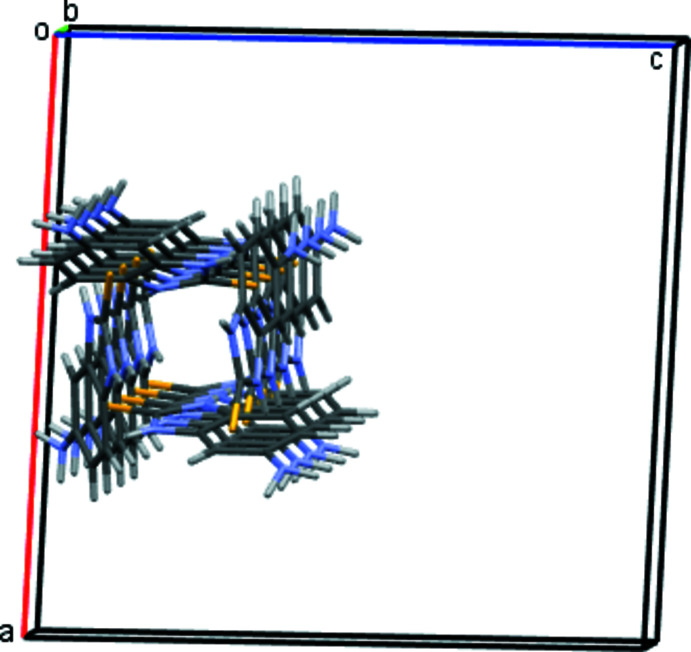
Projection of a tube in the *b*-axis direction.

**Table 1:** Hydrogen-bond geometry (Å, °)

*D*—H⋯*A*	*D*—H	H⋯*A*	*D*⋯*A*	*D*—H⋯*A*
N1*A*—H1*NA*⋯N3*B*	0.80 (3)	2.06 (3)	2.856 (3)	176 (3)
N2*A*—H2*NA*⋯S1*B* ^i^	0.82 (3)	2.54 (3)	3.295 (2)	154 (3)
N3*A*—H3*NA*⋯S1*A* ^ii^	0.84 (4)	2.75 (4)	3.551 (3)	159 (3)
N3*A*—H3*NB*⋯C4*B* ^iii^	0.89 (4)	2.71 (4)	3.483 (4)	145 (3)
N3*A*—H3*NB*⋯C5*B* ^iii^	0.89 (4)	2.81 (4)	3.643 (4)	155 (3)
N1*B*—H1*NB*⋯N3*A* ^iv^	0.88 (3)	2.02 (3)	2.884 (3)	171 (3)
N2*B*—H2*NB*⋯S1*A* ^v^	0.85 (3)	2.56 (3)	3.340 (2)	153 (3)
N3*B*—H3*NC*⋯S1*B* ^vi^	0.86 (3)	2.91 (3)	3.672 (3)	149 (2)
N3*B*—H3*ND*⋯S1*B* ^vii^	0.85 (3)	2.70 (3)	3.477 (3)	153 (3)
C5*A*—H5*A*⋯S1*A* ^viii^	0.96 (3)	2.96 (3)	3.656 (3)	130.2 (19)
C5*B*—H5*B*⋯C1*A* ^ii^	0.90 (3)	2.78 (3)	3.562 (4)	147 (3)

Symmetry codes: (i) -x+1, y+1, -z+{\script{1\over 2}}; (ii) -x+{\script{3\over 2}}, y-{\script{1\over 2}}, -z+{\script{1\over 2}}; (iii) x, -y+1, z+{\script{1\over 2}}; (iv) -x+1, y, -z+{\script{1\over 2}}; (v) x, y-1, z; (vi) -x+1, -y+1, -z; (vii) x+{\script{1\over 2}}, y+{\script{1\over 2}}, z; (viii) x, -y+2, z+{\script{1\over 2}}.

**Table 2:** Experimental details

Crystal data	
Chemical formula	C_7_H_7_N_3_S
*M*_r_	165.22
Crystal system, space group	Monoclinic, *C*2/*c*
Temperature (K)	293
*a*, *b*, *c* (Å)	16.1179 (14), 11.8796 (11), 16.5649 (15)
β (°)	91.974 (8)
*V* (Å^3^)	3169.9 (5)
*Z*	16
Radiation type	Mo *K*α
μ (mm^−1^)	0.34
Crystal size (mm)	0.80 × 0.26 × 0.08

Data collection	
Diffractometer	Xcalibur, Sapphire3
Absorption correction	Multi-scan (*CrysAlis PRO*; Rigaku OD, 2018 ▸)
*T*_min_, *T*_max_	0.370, 1.000
No. of measured, independent and observed [*I* > 2σ(*I*)] reflections	12390, 2787, 2417
*R*_int_	0.079
(sin θ/λ)_max_ (Å^−1^)	0.595

Refinement	
*R*[*F* ^2^ > 2σ(*F* ^2^)], *wR*(*F* ^2^), *S*	0.052, 0.138, 1.05
No. of reflections	2787
No. of parameters	255
H-atom treatment	All H-atom parameters refined
Δρ_max_, Δρ_min_ (e Å^−3^)	0.33, −0.27

Computer programs: *CrysAlis PRO* (Rigaku OD, 2018 ▸), *SHELXT2014/5* (Sheldrick, 2015*a* ▸), *SHELXL2016/6* (Sheldrick, 2015*b* ▸), *Mercury* (Macrae *et al.*, 2020 ▸) and *Olex2* (Dolomanov *et al.*, 2009 ▸).

## supplementary crystallographic information

### Crystal data

**Table:**

C_7_H_7_N_3_S	*F*(000) = 1376
*M**_r_* = 165.22	*D*_x_ = 1.385 Mg m^−^^3^
Monoclinic, *C*2/*c*	Mo *K*α radiation, λ = 0.71073 Å
*a* = 16.1179 (14) Å	Cell parameters from 2937 reflections
*b* = 11.8796 (11) Å	θ = 3.5–26.9°
*c* = 16.5649 (15) Å	µ = 0.34 mm^−^^1^
β = 91.974 (8)°	*T* = 293 K
*V* = 3169.9 (5) Å^3^	Plate, colorless
*Z* = 16	0.80 × 0.26 × 0.08 mm

### Data collection

**Table:**

Xcalibur, Sapphire3 diffractometer	2787 independent reflections
Radiation source: Enhance (Mo) X-ray Source	2417 reflections with *I* > 2σ(*I*)
Detector resolution: 16.1827 pixels mm^-1^	*R*_int_ = 0.079
ω scans	θ_max_ = 25.0°, θ_min_ = 3.2°
Absorption correction: multi-scan (CrysAlisPro; Rigaku OD, 2018)	*h* = −19→18
*T*_min_ = 0.370, *T*_max_ = 1.000	*k* = −14→14
12390 measured reflections	*l* = −19→19

### Refinement

**Table:**

Refinement on *F*^2^	0 restraints
Least-squares matrix: full	Hydrogen site location: difference Fourier map
*R*[*F*^2^ > 2σ(*F*^2^)] = 0.052	All H-atom parameters refined
*wR*(*F*^2^) = 0.138	*w* = 1/[σ^2^(*F*_o_^2^) + (0.0719*P*)^2^ + 1.8442*P*] where *P* = (*F*_o_^2^ + 2*F*_c_^2^)/3
*S* = 1.05	(Δ/σ)_max_ = 0.001
2787 reflections	Δρ_max_ = 0.33 e Å^−^^3^
255 parameters	Δρ_min_ = −0.27 e Å^−^^3^

### Special details

**Table:**

Geometry. All esds (except the esd in the dihedral angle between two l.s. planes) are estimated using the full covariance matrix. The cell esds are taken into account individually in the estimation of esds in distances, angles and torsion angles; correlations between esds in cell parameters are only used when they are defined by crystal symmetry. An approximate (isotropic) treatment of cell esds is used for estimating esds involving l.s. planes.

### Fractional atomic coordinates and isotropic or equivalent isotropic displacement parameters (Å^2^)

**Table:**

	*x*	*y*	*z*	*U*_iso_*/*U*_eq_	
S1B	0.37352 (5)	0.24474 (6)	0.14471 (5)	0.0546 (3)	
N1B	0.44866 (14)	0.44339 (17)	0.11231 (14)	0.0388 (5)	
H1NB	0.402 (2)	0.477 (3)	0.098 (2)	0.060 (9)*	
N2B	0.53394 (13)	0.31429 (19)	0.15625 (14)	0.0406 (5)	
H2NB	0.5498 (18)	0.248 (2)	0.1694 (18)	0.048 (8)*	
N3B	0.66860 (17)	0.7248 (2)	0.07344 (15)	0.0425 (6)	
H3NC	0.6461 (19)	0.754 (2)	0.031 (2)	0.047 (9)*	
H3ND	0.721 (2)	0.729 (2)	0.0726 (18)	0.049 (9)*	
C1B	0.52713 (14)	0.4917 (2)	0.11265 (14)	0.0345 (6)	
C2B	0.55387 (16)	0.5967 (2)	0.08966 (15)	0.0365 (6)	
H2B	0.5159 (17)	0.655 (2)	0.0704 (16)	0.049 (8)*	
C3B	0.63884 (15)	0.6175 (2)	0.09525 (14)	0.0360 (6)	
C4B	0.69335 (17)	0.5355 (2)	0.12677 (17)	0.0428 (6)	
H4B	0.748 (2)	0.556 (2)	0.1314 (18)	0.051 (8)*	
C5B	0.66596 (17)	0.4308 (2)	0.15038 (18)	0.0449 (7)	
H5B	0.699 (2)	0.378 (3)	0.174 (2)	0.063 (9)*	
C6B	0.58190 (15)	0.4093 (2)	0.14187 (15)	0.0365 (6)	
C7B	0.45329 (16)	0.3353 (2)	0.13759 (15)	0.0382 (6)	
S1A	0.60205 (5)	1.05022 (6)	0.13822 (4)	0.0479 (3)	
N1A	0.63692 (14)	0.85370 (18)	0.21461 (13)	0.0397 (5)	
H1NA	0.6481 (17)	0.819 (3)	0.1755 (18)	0.044 (8)*	
N2A	0.59495 (13)	0.9834 (2)	0.29505 (13)	0.0383 (5)	
H2NA	0.5855 (18)	1.049 (3)	0.3087 (18)	0.046 (8)*	
N3A	0.69384 (16)	0.57892 (19)	0.43321 (16)	0.0420 (6)	
H3NA	0.737 (2)	0.556 (3)	0.411 (2)	0.061 (11)*	
H3NB	0.705 (2)	0.580 (3)	0.486 (2)	0.067 (10)*	
C1A	0.63986 (15)	0.8070 (2)	0.29109 (14)	0.0343 (6)	
C2A	0.66733 (16)	0.7028 (2)	0.31939 (16)	0.0382 (6)	
H2A	0.6856 (16)	0.644 (2)	0.2830 (17)	0.044 (7)*	
C3A	0.66781 (15)	0.6855 (2)	0.40200 (15)	0.0354 (6)	
C4A	0.63934 (17)	0.7692 (2)	0.45370 (17)	0.0421 (6)	
H4A	0.6398 (18)	0.753 (2)	0.5104 (19)	0.050 (8)*	
C5A	0.61082 (18)	0.8722 (2)	0.42509 (16)	0.0432 (6)	
H5A	0.5872 (16)	0.927 (2)	0.4604 (16)	0.039 (7)*	
C6A	0.61262 (15)	0.8897 (2)	0.34291 (15)	0.0344 (5)	
C7A	0.61113 (15)	0.9613 (2)	0.21741 (15)	0.0373 (6)	

### Atomic displacement parameters (Å^2^)

**Table:**

	*U*^11^	*U*^22^	*U*^33^	*U*^12^	*U*^13^	*U*^23^
S1B	0.0502 (5)	0.0400 (4)	0.0723 (6)	−0.0110 (3)	−0.0143 (4)	0.0145 (3)
N1B	0.0347 (12)	0.0318 (11)	0.0493 (13)	0.0011 (9)	−0.0089 (10)	0.0041 (10)
N2B	0.0421 (12)	0.0278 (12)	0.0514 (13)	0.0040 (9)	−0.0072 (10)	0.0060 (10)
N3B	0.0432 (14)	0.0435 (14)	0.0408 (13)	−0.0074 (11)	0.0015 (11)	0.0010 (11)
C1B	0.0343 (13)	0.0337 (13)	0.0350 (12)	0.0000 (10)	−0.0058 (10)	−0.0007 (10)
C2B	0.0388 (14)	0.0304 (13)	0.0399 (13)	0.0032 (11)	−0.0035 (11)	0.0005 (11)
C3B	0.0421 (14)	0.0340 (13)	0.0320 (12)	−0.0014 (11)	0.0004 (11)	−0.0065 (10)
C4B	0.0343 (14)	0.0460 (16)	0.0477 (15)	0.0009 (12)	−0.0031 (12)	−0.0043 (12)
C5B	0.0383 (15)	0.0410 (15)	0.0546 (16)	0.0082 (12)	−0.0109 (13)	0.0001 (13)
C6B	0.0379 (13)	0.0321 (13)	0.0391 (13)	0.0036 (11)	−0.0056 (11)	0.0005 (11)
C7B	0.0439 (14)	0.0322 (13)	0.0379 (13)	−0.0004 (11)	−0.0069 (11)	0.0027 (11)
S1A	0.0595 (5)	0.0385 (4)	0.0459 (4)	0.0055 (3)	0.0033 (3)	0.0090 (3)
N1A	0.0565 (14)	0.0307 (12)	0.0322 (11)	0.0023 (10)	0.0034 (10)	−0.0023 (10)
N2A	0.0462 (13)	0.0302 (12)	0.0384 (12)	0.0063 (10)	0.0006 (10)	−0.0047 (10)
N3A	0.0443 (14)	0.0378 (13)	0.0432 (13)	−0.0024 (10)	−0.0074 (12)	0.0044 (11)
C1A	0.0376 (13)	0.0309 (13)	0.0342 (12)	−0.0024 (10)	−0.0023 (10)	−0.0008 (10)
C2A	0.0455 (15)	0.0297 (13)	0.0392 (14)	−0.0013 (11)	−0.0011 (11)	−0.0038 (11)
C3A	0.0358 (13)	0.0318 (13)	0.0383 (13)	−0.0060 (10)	−0.0054 (10)	0.0000 (11)
C4A	0.0474 (15)	0.0447 (15)	0.0338 (14)	−0.0055 (12)	−0.0048 (12)	0.0008 (12)
C5A	0.0542 (16)	0.0397 (15)	0.0354 (14)	0.0016 (12)	−0.0008 (12)	−0.0068 (12)
C6A	0.0358 (13)	0.0305 (13)	0.0368 (13)	0.0002 (10)	−0.0024 (10)	−0.0014 (10)
C7A	0.0355 (13)	0.0330 (13)	0.0430 (15)	−0.0003 (10)	−0.0017 (11)	−0.0011 (11)

### Geometric parameters (Å, º)

**Table:**

S1B—C7B	1.684 (3)	S1A—C7A	1.686 (3)
N1B—C7B	1.351 (3)	N1A—C7A	1.346 (3)
N1B—C1B	1.389 (3)	N1A—C1A	1.382 (3)
N1B—H1NB	0.88 (3)	N1A—H1NA	0.80 (3)
N2B—C7B	1.349 (3)	N2A—C7A	1.347 (3)
N2B—C6B	1.393 (3)	N2A—C6A	1.390 (3)
N2B—H2NB	0.85 (3)	N2A—H2NA	0.82 (3)
N3B—C3B	1.413 (3)	N3A—C3A	1.426 (3)
N3B—H3NC	0.86 (3)	N3A—H3NA	0.84 (4)
N3B—H3ND	0.85 (3)	N3A—H3NB	0.89 (4)
C1B—C2B	1.378 (4)	C1A—C6A	1.386 (3)
C1B—C6B	1.394 (3)	C1A—C2A	1.390 (4)
C2B—C3B	1.391 (4)	C2A—C3A	1.384 (3)
C2B—H2B	0.97 (3)	C2A—H2A	0.98 (3)
C3B—C4B	1.401 (4)	C3A—C4A	1.400 (4)
C4B—C5B	1.380 (4)	C4A—C5A	1.385 (4)
C4B—H4B	0.92 (3)	C4A—H4A	0.96 (3)
C5B—C6B	1.381 (4)	C5A—C6A	1.378 (4)
C5B—H5B	0.90 (3)	C5A—H5A	0.96 (3)
			
C7B—N1B—C1B	110.5 (2)	C7A—N1A—C1A	110.5 (2)
C7B—N1B—H1NB	124 (2)	C7A—N1A—H1NA	127 (2)
C1B—N1B—H1NB	125 (2)	C1A—N1A—H1NA	122 (2)
C7B—N2B—C6B	110.3 (2)	C7A—N2A—C6A	110.3 (2)
C7B—N2B—H2NB	121 (2)	C7A—N2A—H2NA	119 (2)
C6B—N2B—H2NB	129 (2)	C6A—N2A—H2NA	129 (2)
C3B—N3B—H3NC	116 (2)	C3A—N3A—H3NA	111 (2)
C3B—N3B—H3ND	114 (2)	C3A—N3A—H3NB	113 (2)
H3NC—N3B—H3ND	111 (3)	H3NA—N3A—H3NB	108 (3)
C2B—C1B—N1B	131.8 (2)	N1A—C1A—C6A	106.3 (2)
C2B—C1B—C6B	122.1 (2)	N1A—C1A—C2A	131.8 (2)
N1B—C1B—C6B	106.1 (2)	C6A—C1A—C2A	121.8 (2)
C1B—C2B—C3B	117.3 (2)	C3A—C2A—C1A	117.2 (2)
C1B—C2B—H2B	122.3 (17)	C3A—C2A—H2A	120.9 (16)
C3B—C2B—H2B	120.4 (17)	C1A—C2A—H2A	122.0 (16)
C2B—C3B—C4B	120.3 (2)	C2A—C3A—C4A	120.6 (2)
C2B—C3B—N3B	119.0 (2)	C2A—C3A—N3A	118.8 (2)
C4B—C3B—N3B	120.6 (2)	C4A—C3A—N3A	120.5 (2)
C5B—C4B—C3B	122.0 (3)	C5A—C4A—C3A	122.0 (3)
C5B—C4B—H4B	122.1 (18)	C5A—C4A—H4A	120.0 (17)
C3B—C4B—H4B	115.9 (18)	C3A—C4A—H4A	118.0 (17)
C4B—C5B—C6B	117.4 (3)	C6A—C5A—C4A	117.0 (3)
C4B—C5B—H5B	124 (2)	C6A—C5A—H5A	121.3 (16)
C6B—C5B—H5B	119 (2)	C4A—C5A—H5A	121.6 (16)
C5B—C6B—N2B	132.9 (2)	C5A—C6A—C1A	121.5 (2)
C5B—C6B—C1B	120.8 (2)	C5A—C6A—N2A	132.3 (2)
N2B—C6B—C1B	106.3 (2)	C1A—C6A—N2A	106.1 (2)
N2B—C7B—N1B	106.8 (2)	N1A—C7A—N2A	106.7 (2)
N2B—C7B—S1B	126.69 (19)	N1A—C7A—S1A	125.9 (2)
N1B—C7B—S1B	126.5 (2)	N2A—C7A—S1A	127.3 (2)
			
C7B—N1B—C1B—C2B	177.4 (3)	C7A—N1A—C1A—C6A	1.4 (3)
C7B—N1B—C1B—C6B	−1.7 (3)	C7A—N1A—C1A—C2A	−175.1 (3)
N1B—C1B—C2B—C3B	−177.8 (2)	N1A—C1A—C2A—C3A	174.9 (3)
C6B—C1B—C2B—C3B	1.2 (4)	C6A—C1A—C2A—C3A	−1.1 (4)
C1B—C2B—C3B—C4B	−2.9 (4)	C1A—C2A—C3A—C4A	1.8 (4)
C1B—C2B—C3B—N3B	−179.0 (2)	C1A—C2A—C3A—N3A	178.6 (2)
C2B—C3B—C4B—C5B	2.4 (4)	C2A—C3A—C4A—C5A	−0.8 (4)
N3B—C3B—C4B—C5B	178.5 (3)	N3A—C3A—C4A—C5A	−177.6 (2)
C3B—C4B—C5B—C6B	0.0 (4)	C3A—C4A—C5A—C6A	−0.8 (4)
C4B—C5B—C6B—N2B	177.3 (3)	C4A—C5A—C6A—C1A	1.5 (4)
C4B—C5B—C6B—C1B	−1.8 (4)	C4A—C5A—C6A—N2A	−174.7 (3)
C7B—N2B—C6B—C5B	−179.4 (3)	N1A—C1A—C6A—C5A	−177.5 (2)
C7B—N2B—C6B—C1B	−0.3 (3)	C2A—C1A—C6A—C5A	−0.6 (4)
C2B—C1B—C6B—C5B	1.2 (4)	N1A—C1A—C6A—N2A	−0.4 (3)
N1B—C1B—C6B—C5B	−179.6 (2)	C2A—C1A—C6A—N2A	176.5 (2)
C2B—C1B—C6B—N2B	−178.1 (2)	C7A—N2A—C6A—C5A	176.0 (3)
N1B—C1B—C6B—N2B	1.2 (3)	C7A—N2A—C6A—C1A	−0.7 (3)
C6B—N2B—C7B—N1B	−0.8 (3)	C1A—N1A—C7A—N2A	−1.8 (3)
C6B—N2B—C7B—S1B	179.5 (2)	C1A—N1A—C7A—S1A	177.73 (19)
C1B—N1B—C7B—N2B	1.5 (3)	C6A—N2A—C7A—N1A	1.6 (3)
C1B—N1B—C7B—S1B	−178.68 (19)	C6A—N2A—C7A—S1A	−177.99 (19)

### Hydrogen-bond geometry (Å, º)

**Table:**

*D*—H···*A*	*D*—H	H···*A*	*D*···*A*	*D*—H···*A*
N1*A*—H1*NA*···N3*B*	0.80 (3)	2.06 (3)	2.856 (3)	176 (3)
N2*A*—H2*NA*···S1*B*^i^	0.82 (3)	2.54 (3)	3.295 (2)	154 (3)
N3*A*—H3*NA*···S1*A*^ii^	0.84 (4)	2.75 (4)	3.551 (3)	159 (3)
N3*A*—H3*NB*···C4*B*^iii^	0.89 (4)	2.71 (4)	3.483 (4)	145 (3)
N3*A*—H3*NB*···C5*B*^iii^	0.89 (4)	2.81 (4)	3.643 (4)	155 (3)
N1*B*—H1*NB*···N3*A*^iv^	0.88 (3)	2.02 (3)	2.884 (3)	171 (3)
N2*B*—H2*NB*···S1*A*^v^	0.85 (3)	2.56 (3)	3.340 (2)	153 (3)
N3*B*—H3*NC*···S1*B*^vi^	0.86 (3)	2.91 (3)	3.672 (3)	149 (2)
N3*B*—H3*ND*···S1*B*^vii^	0.85 (3)	2.70 (3)	3.477 (3)	153 (3)
C5*A*—H5*A*···S1*A*^viii^	0.96 (3)	2.96 (3)	3.656 (3)	130.2 (19)
C5*B*—H5*B*···C1*A*^ii^	0.90 (3)	2.78 (3)	3.562 (4)	147 (3)

Symmetry codes: (i) −*x*+1, *y*+1, −*z*+1/2; (ii) −*x*+3/2, *y*−1/2, −*z*+1/2; (iii) *x*, −*y*+1, *z*+1/2; (iv) −*x*+1, *y*, −*z*+1/2; (v) *x*, *y*−1, *z*; (vi) −*x*+1, −*y*+1, −*z*; (vii) *x*+1/2, *y*+1/2, *z*; (viii) *x*, −*y*+2, *z*+1/2.
